# Anesthesia Management in Pregnant Women with Cardiac Disease: A Narrative Review

**DOI:** 10.5152/eurasianjmed.2026.261693

**Published:** 2026-10-16

**Authors:** Dudu Berrin Gunaydin, Nuray Camgoz Eryilmaz, Gozde Inan

**Affiliations:** 1 Department of Anesthesiology and Reanimation, Gazi University Faculty of Medicine, Ankara, Türkiye

**Keywords:** Anesthesia, cardiac, delivery, pregnancy

## Abstract

This review addresses the perspective of an obstetric anesthesiologist, focusing on the practical principles of peripartum anesthetic management by integrating current evidence with contemporary guideline recommendations rather than providing a comprehensive overview of cardiovascular disease during pregnancy. It also provides a clinically oriented approach to hemodynamic optimization, delivery planning, neuraxial and general anesthetic management, and the treatment of obstetric cardiac emergencies. Ultimately, this review aims to answer the question every anesthesiologist faces in daily practice: *“What should the anaesthesiologist do for cardiac pregnant patient?”*

**Main Points**

- A multidisciplinary model of care centered on the Pregnancy Heart Team includes cardiologists, maternal–fetal medicine specialists, obstetricians, anesthesiologists, and other specialists according to the underlying cardiac lesion
- Management extends far beyond the choice of anesthetic technique to the safe peri-partum care
- Thorough understanding of pregnancy-related cardiovascular physiology, lesion-specific hemodynamics, maternal risk assessment, hemodynamic monitoring, and the anticipated cardiovascular consequences of labor, delivery, and the immediate postpartum period is required
- Practical aspects of anesthetic management, including early risk stratification, collaborative management, meticulous hemodynamic optimization, and individualized anesthetic planning, are fundamental to improve maternal and neonatal outcomes

## Introduction

Cardiovascular disease (CVD) remains 1 of the leading causes of pregnancy-related maternal morbidity and mortality worldwide, accounting for more than one-quarter of maternal deaths. Hypertensive disorders complicate approximately 5%-10% of all pregnancies and represent the most common cardiovascular conditions during pregnancy. In Europe, congenital heart disease (CHD) accounts for 75%-82% of pregnancy-associated CVD, whereas rheumatic valvular heart disease continues to predominate in many low- and middle-income countries, representing 56%-89% of CVD during pregnancy.^1^^,^^2^

Advances in pediatric cardiology and cardiac surgery have enabled increasing numbers of women with complex congenital heart disease to reach reproductive age. Consequently, the prevalence of pregnant women with CHD increased from 6.4 to 9.0 per 10 000 deliveries between 2000 and 2010. Although maternal outcomes associated with congenital heart disease have remained relatively stable in high-income countries, pregnancy-related complications resulting from acquired cardiovascular disease, including heart failure, myocardial infarction, cardiac arrhythmias, and aortic dissection, have become increasingly frequent, particularly during the early postpartum period.^3^ For the anesthesiologist, distinguishing normal physiological adaptations of pregnancy from CVD is clinically significant. Symptoms such as tachycardia, dyspnea, peripheral edema, and exercise intolerance commonly occur during uncomplicated pregnancy but may also be the first manifestations of underlying cardiac pathology. Failure to recognize these warning signs may delay diagnosis, increase the risk of maternal cardiovascular decompensation, and adversely affect both maternal and fetal outcomes.^3^

From the anaesthesiologist’s perspective, management extends far beyond the choice of anesthetic technique. Safe peri-partum care requires a thorough understanding of pregnancy-related cardiovascular physiology, lesion-specific hemodynamics, maternal risk assessment, hemodynamic monitoring, and the anticipated cardiovascular consequences of labor, delivery, and the immediate postpartum period.^1^^-^^3^ This review focuses on the practical aspects of anesthetic management in pregnant women with common CVD, emphasizing contemporary evidence and multidisciplinary strategies that optimize maternal and fetal outcomes. This study used generative AI to improve language, edit grammar, generate content, during the preparation of the manuscript. However, all content of the study has been reviewed and edited by the authors following the use of AI/LLM tools.

## Cardiovascular Physiology and Adaptations to Pregnancy

Pregnancy induces major cardiovascular adaptations that support the increasing metabolic demands of the mother and fetus. Cardiac output (CO), plasma volume, and heart rate (HR) progressively increase, whereas systemic vascular resistance (SVR) and blood pressure (BP) decrease during early pregnancy before returning toward baseline near term (Figure 1). During labor and immediately after delivery, uterine contractions, autotransfusion, and relief of aortocaval compression result in rapid hemodynamic fluctuations and increased venous return, making this a particularly vulnerable period for women with underlying cardiovascular disease. Pregnancy is also associated with physiological cardiac remodeling, including mild ventricular enlargement, increased ventricular mass, and trivial valvular regurgitation, as well as reduced colloid oncotic pressure, which together increase susceptibility to pulmonary edema in the presence of cardiac dysfunction, preeclampsia, or excessive fluid administration. Most cardiovascular adaptations gradually resolve within several months postpartum.^1^^-^^5^

### Cardiovascular Assessment During Pregnancy

Accurate cardiovascular assessment is essential to optimize maternal and fetal outcomes in women with known or suspected CVD. Because normal physiological adaptations of pregnancy often mimic cardiac disease, distinguishing physiological from pathological findings requires careful clinical evaluation.^2^^,^^3^

#### Clinical Assessment

Assessment begins with a detailed history and physical examination. Symptoms such as progressive dyspnea, orthopnea, chest pain, palpitations, syncope, or unexplained fatigue should not be attributed solely to pregnancy. Clinical evaluation should include HR and rhythm, BP, oxygen saturation, signs of heart failure, cardiac murmurs, jugular venous pressure, and peripheral edema. While mild systolic murmurs and dependent edema are common in normal pregnancy, diastolic murmurs, persistent tachycardia, cyanosis, or pulmonary congestion require prompt cardiology assessment. For the anesthesiologist, pre-delivery evaluation should define lesion-specific hemodynamic goals, identify the need for invasive monitoring, and facilitate multidisciplinary peripartum planning.^3^ 5 This study used generative AI to improve language, edit grammar, generate content, during the preparation of the manuscript. However, all content of the study has been reviewed and edited by the authors afterward. 

#### Cardiac Biomarkers

Cardiac biomarkers complement clinical assessment in selected patients. Brain natriuretic peptide (BNP) and N-terminal pro–B-type natriuretic peptide (NT-proBNP) remain within normal limits during uncomplicated pregnancy; elevated levels suggest ventricular dysfunction or heart failure and are particularly useful for monitoring women with congenital heart disease, cardiomyopathy, or impaired ventricular function. Cardiac troponins remain the biomarkers of choice for suspected myocardial injury, and the diagnosis of acute coronary syndrome follows the same principles as in non-pregnant patients. D-dimer levels increase physiologically throughout pregnancy and should therefore be interpreted using pregnancy-adapted diagnostic algorithms rather than as an isolated test.^3^^,^^5^^,^^6^

#### Cardiovascular Imaging

A 12-lead electrocardiogram (ECG) is recommended in women presenting with chest pain, dyspnea, palpitations, or syncope. Mild left-axis deviation and nonspecific ST–T changes may occur during normal pregnancy, whereas Holter monitoring is indicated when intermittent arrhythmias are suspected.^2^^,^^4^

Transthoracic echocardiography (TTE) is the first-line imaging modality for evaluating structural heart disease because it is non-invasive and free of ionizing radiation. It is indicated in women with congenital or valvular heart disease, cardiomyopathy, pulmonary hypertension, aortic disease, or unexplained hypoxemia. Mild chamber enlargement and physiological valvular regurgitation are common findings in late pregnancy.^2^^-^^4^

Exercise testing is mainly performed before conception to assess functional capacity and maternal risk in women with congenital or valvular heart disease. During pregnancy, chest radiography may be performed when clinically indicated because fetal radiation exposure is minimal, whereas lung ultrasound provides a rapid bedside assessment of pulmonary congestion. Computed tomography (CT) should be reserved for selected cases such as suspected pulmonary embolism or acute aortic syndrome. Cardiac magnetic resonance imaging (MRI) offers detailed evaluation of ventricular function, congenital heart disease, and aortic pathology without ionizing radiation when echocardiography is inconclusive.^2^^-^^5^

## Risk Assessment for Cardiovascular Complications During Pregnancy

Accurate maternal cardiovascular risk stratification is essential for counseling, antenatal surveillance, delivery planning, and peri-partum anesthetic management. Ideally, risk assessment should begin before conception, allowing optimization of maternal cardiovascular status, review of cardiac medications, and discussion of pregnancy-related risks. In women with previously unrecognized CVD, early diagnosis during pregnancy remains critical for preventing maternal decompensation and improving both maternal and fetal outcomes.^1^^-^^3^

Preconception evaluation should include a comprehensive cardiovascular assessment comprising electrocardiography (ECG), TTE, and, when indicated, exercise stress testing. Additional imaging with CT or cardiac MRI should be considered in women with suspected aortic pathology or complex congenital heart disease. Maternal exercise capacity, particularly peak oxygen consumption and the ability to achieve more than 80% of the predicted maximal exercise capacity, has been associated with more favorable maternal outcomes during pregnancy.^2^ There are several validated tools to assist clinicians in estimating maternal cardiovascular risk and planning appropriate surveillance throughout pregnancy.^3^

### Canadian Cardiac Disease in Pregnancy Risk Index II

Canadian Cardiac Disease in Pregnancy Risk Index II (CARPREG II) is a comprehensive risk prediction model incorporating lesion-specific characteristics, maternal clinical variables, and healthcare-related factors to estimate the likelihood of maternal cardiac complications during pregnancy. It is widely used to support individualized counseling and multidisciplinary planning.^7^

### Zwangerschap bij Aangeboren HARtAfwijkingen

The Zwangerschap bij Aangeboren HARtAfwijkingen (ZAHARA) score (pregnancy in women with congenital heart disease – maternal cardiovascular risk prediction score) was developed specifically for women with congenital heart disease and incorporates weighted lesion-specific variables to estimate the risk of maternal cardiovascular complications. Although primarily validated in congenital heart disease, it provides valuable complementary information when counseling affected women.^8^

## Modified World Health Organization Maternal Cardiovascular Risk Classification

The modified World Health Organization (mWHO) classification remains the most widely adopted in contemporary clinical practice and forms the basis of the 2023 European Society of Cardiology Guidelines (Table 1). Rather than generating a numerical score, the mWHO classification categorizes women according to their underlying cardiac condition into 4 risk classes (I-IV), reflecting progressively increasing risks of maternal morbidity and mortality. Women classified as mWHO Class I have little or no increase in maternal cardiovascular risk and generally require only limited cardiology follow-up during pregnancy. At the opposite end of the spectrum, mWHO Class IV includes conditions associated with an extremely high risk of maternal mortality or severe cardiovascular morbidity. Pregnancy is generally contraindicated in these patients, and if pregnancy occurs, management should be undertaken in highly specialized tertiary referral centers with immediate access to advanced maternal, cardiac, and neonatal services.^3^

Patients classified as mWHO Classes III and IV require comprehensive multidisciplinary care coordination. In addition to specialist cardiology and maternal-fetal medicine services, delivery should be planned in centers with dedicated obstetric anesthesia, intensive care, interventional cardiology, cardiac surgery, and mechanical circulatory support capabilities whenever appropriate.^3^

Importantly, although the mWHO classification provides an excellent framework for estimating maternal cardiovascular risk, it should not replace individualized clinical judgment. Anesthetic planning must also incorporate lesion-specific hemodynamics, ventricular function, functional capacity, anticipated cardiovascular responses to labor or Caesarean delivery, and the expertise and resources available within the treating institution. Consequently, risk prediction models should be regarded as complementary tools that support, rather than replace, multidisciplinary decision-making.^3^

## Pre-anesthetic Assessment

Successful anesthetic management of pregnant women with CVD begins long before labor or Caesarean delivery. Comprehensive pre-anesthetic assessment identifies maternal cardiovascular risk, establishes lesion-specific hemodynamic goals, anticipates potential peri-partum complications, and develops an individualized management plan. Ultimately, optimal maternal and fetal outcomes depend on accurate cardiovascular risk stratification, patient education, and coordinated multidisciplinary care.^1^

## The Pregnancy Heart Team

Pregnancy Heart Team includes an obstetrician, maternal-fetal medicine specialist, cardiologist, and obstetric anesthesiologist. Depending on the underlying cardiac lesion, additional specialists including cardiac surgeons, cardiac electrophysiologists, pulmonary hypertension experts, interventional cardiologists, neonatologists, intensivists, geneticists, clinical pharmacists, and mental health professionals may contribute to patient care. By integrating cardiovascular physiology with obstetric and anesthetic considerations, obstetric anesthesiologists play a pivotal role within the team (Table 2).^1^^,^^9^

## Delivery Management

Delivery planning should begin early during pregnancy and be revisited whenever maternal cardiovascular status changes. The timing, place, and mode of delivery should be individualized according to maternal cardiovascular condition, fetal well-being, obstetric considerations, and institutional resources. For women with low-risk cardiac lesions (mWHO Class II), delivery may be safely coordinated by maternal-fetal medicine specialists and cardiologists in appropriately equipped local hospitals. In contrast, women classified as mWHO Classes III and IV require delivery in tertiary referral centers with immediate access to comprehensive multidisciplinary cardiovascular and obstetric services.^3^^,^^10^

### Timing of Delivery

The optimal timing of delivery should balance the risks associated with prematurity against the maternal and fetal benefits of continuing pregnancy. Decisions should be individualized following multidisciplinary discussion. For women with stable cardiovascular disease who have not entered spontaneous labor and have no indication for earlier delivery, the American College of Obstetricians and Gynecologists (ACOG) recommends elective induction of labor between 39 and 40 weeks’ gestation. Although evidence specific to women with cardiovascular disease remains limited, planned induction may reduce Cesarean delivery rates, facilitate multidisciplinary coordination, and allow delivery to occur under optimal maternal cardiovascular conditions. Commonly used induction agents are generally considered safe in women with cardiovascular disease. When maternal cardiac surgery becomes necessary during pregnancy, timing requires careful consideration. Although cardiac surgery before fetal viability carries substantial fetal risk, outcomes improve significantly once fetal viability has been achieved. Whenever maternal condition permits, delivery after fetal viability and before cardiac surgery offers the greatest opportunity to optimize both maternal and neonatal outcomes.^1^

### Mode of Delivery

For most women with CVD, vaginal delivery remains the preferred mode of delivery. Compared with cesarean delivery, vaginal birth is associated with lower risks of hemorrhage, infection, thromboembolic complications, and postoperative recovery, while allowing more gradual hemodynamic changes that are generally better tolerated by the maternal cardiovascular system. Consequently, contemporary international guidelines recommend vaginal delivery whenever there are no obstetric or cardiac contraindications.^1^^,^^9^

For women whose planned place of delivery is a tertiary referral center located distant from their residence, elective induction of labor may facilitate coordinated multidisciplinary care. Planned admission to ensure the availability of appropriate personnel, monitoring, blood products, cardiovascular support, and neonatal services while minimizing the likelihood of unplanned emergency interventions.^11^

The delivery strategy should always be individualized according to the underlying cardiac lesion, maternal functional status, fetal well-being, and obstetric considerations. Before labor, whether spontaneous vaginal delivery is appropriate to determine the anticipated hemodynamic goals during labor, and/or decide operative assistance during the second stage is likely to be required. Although the Valsalva maneuver during the second stage of labor is well tolerated by most women with CVD, prolonged or forceful maternal pushing may be poorly tolerated in selected high-risk conditions. Patients with significant ventricular dysfunction, Fontan circulation, severe pulmonary hypertension, or markedly reduced cardiovascular reserve may benefit from passive fetal descent followed by assisted vaginal delivery using forceps or vacuum extraction once the fetal head reaches the pelvic floor. This strategy minimizes maternal exertion while preserving the advantages of vaginal birth.^1^^,^^5^^,^^9^

The hemodynamic consequences of the Valsalva maneuver deserve particular consideration. First, the marked reduction in venous return during forceful pushing may significantly decrease preload and CO in women with preload-dependent lesions such as severe mitral stenosis, severe aortic stenosis, or hypertrophic obstructive cardiomyopathy. Second, restoration of ventricular filling followed by vigorous left ventricular contraction generates substantial aortic wall shear stress, potentially increasing the risk of aortic complications in women with underlying aortopathy. Recognition of these physiological principles is essential when planning the conduct of labor in women with complex cardiovascular disease. Cesarean delivery should therefore be reserved primarily for conventional obstetric indications, including breech presentation, failure to progress during labor, or non-reassuring fetal status. However, several cardiovascular conditions also warrant planned Cesarean delivery. These include women receiving therapeutic warfarin at the onset of labor because of the risk of fetal intracranial hemorrhage, acute or chronic aortic dissection, and selected high-risk aortopathies, including bicuspid aortic valve with an ascending aortic diameter exceeding 5 cm, Turner syndrome with an aortic size index greater than 2.5 cm/m^2^, Marfan or Loeys–Dietz syndrome with an aortic diameter exceeding 4.5 cm, and vascular Ehlers–Danlos syndrome. Cesarean delivery should also be considered in women with refractory heart failure, maternal cardiogenic shock, or other conditions in which the physiological demands of labor are unlikely to be tolerated.^12^^,^^14^

In selected patients, Caesarean delivery may be coordinated with cardiovascular interventions such as peripartum balloon valvuloplasty, initiation of mechanical circulatory support, or planned cardiac surgery. Such coordinated multidisciplinary management optimizes procedural timing, maintains hemodynamic stability, and ensures immediate access to specialized cardiovascular resources should maternal deterioration occur.^13^

## Peripartum Plan

The intrapartum and early postpartum periods represent the greatest hemodynamic challenge in pregnancy because uterine contractions, delivery, autotransfusion, and postpartum fluid shifts substantially increase cardiovascular workload. Anesthetic management should preserve adequate preload and afterload, maintain sinus rhythm, avoid significant HR fluctuations, and optimize myocardial function according to the underlying cardiac lesion.^1^^,^^5^

### Hemodynamic Goals

Peripartum management should focus on lesion-specific hemodynamic targets while anticipating cardiovascular instability before decompensation occurs. Close communication within the multidisciplinary team is essential to optimize maternal and fetal outcomes.^15^

### Preload Considerations

Women with impaired ventricular function or preload-dependent lesions are particularly vulnerable to heart failure during labor and immediately after delivery because of autotransfusion and rapid hemodynamic shifts. Careful fluid management is therefore essential, with early recognition and treatment of pulmonary edema when necessary. Maintenance of sinus rhythm is particularly important in patients with diastolic dysfunction or preload-dependent lesions. Early neuraxial labor analgesia helps reduce pain-induced sympathetic activation, thereby limiting tachycardia and improving hemodynamic stability.^15^^,^^16^

### Afterload Considerations

Adequate SVR should be maintained in women with afterload-dependent lesions, particularly severe left ventricular outflow tract obstruction. Sudden reductions in afterload caused by neuraxial anesthesia, hemorrhage, or rapid intravenous oxytocin may impair coronary perfusion and precipitate cardiovascular collapse. Hypotension should be treated promptly with carefully titrated vasopressors while restoring intravascular volume, and sympathetic blockade should be introduced gradually during neuraxial anesthesia.^15^

### Hemodynamic Monitoring

The intensity of maternal hemodynamic monitoring should be individualized according to the severity of the underlying cardiac lesion, anticipated hemodynamic instability, and mode of delivery. Standard monitoring is adequate for most women with low-risk CVD, whereas invasive arterial pressure monitoring should be considered in patients with severe valvular disease, significant ventricular dysfunction, pulmonary hypertension, complex congenital heart disease, or when major hemodynamic fluctuations are anticipated.^15^^,^^17^

Central venous catheterization is not used routinely but may be helpful when vasoactive drug administration or prolonged hemodynamic support is expected. Pulmonary artery catheterization is rarely required and should be reserved for highly selected patients with severe pulmonary hypertension or refractory heart failure in experienced centers. Bedside TTE or transesophageal echocardiogram (TEE) may provide valuable real-time assessment of ventricular filling, contractility, and volume status during acute cardiovascular deterioration.^18^^,^^19^

## Anesthetic and Analgesic Management

### Neuraxial Analgesia for Labor

Early neuraxial labor analgesia is recommended for most women with CVD because it attenuates pain-induced sympathetic activation, limits tachycardia and hypertension, and reduces myocardial oxygen consumption. Epidural analgesia using incremental doses of dilute local anesthetic combined with opioids provides excellent analgesia while minimizing abrupt hemodynamic changes. Sudden sympathetic blockade should be avoided, particularly in women with fixed CO lesions or severe ventricular dysfunction. Neuraxial labor analgesia practice is summarized in box 1.^1^^,^^15^^,^^16^^,^^20^^-^^22^

### Neuraxial Anesthesia for Caesarean Delivery

Neuraxial anesthesia remains the preferred technique for Caesarean delivery including for patients with mWHO class III or IV lesions because it avoids airway manipulation, preserves spontaneous ventilation, and is associated with lower maternal morbidity than general anesthesia. Gradual establishment of sympathetic blockade using titrated epidural anesthesia or combined spinal epidural (CSE) techniques is generally better tolerated. Single-shot spinal anesthesia is contraindicated in women at risk of hemodynamic instability. The choice of anesthetic technique should be individualized to the patient with the anesthesiologist. Practice of neuraxial anesthesia is summarized in box 2.^1^^,^^15^^,^^17^^,^^23^^-^^26^

### General Anesthesia for Caesarean Delivery

Although neuraxial anesthesia remains the preferred technique for Caesarean delivery in most women with cardiovascular disease, general anesthesia is indicated in selected clinical situations where maternal hemodynamic stability or procedural safety cannot be ensured with neuraxial techniques. General anesthesia should be reserved for standard obstetric indications or when neuraxial techniques are contraindicated or unlikely to provide adequate cardiovascular stability. Careful induction with hemodynamically stable agents, avoidance of significant fluctuations in heart rate and blood pressure, and meticulous airway management are essential. Practice of general anesthesia is summarized in box 3.^15^^,^^17^^,^^27^^-^^31^

## Lesion Specific Considerations

Anesthetic management should always be guided by lesion-specific cardiovascular physiology. Practical aspects of anesthetic management in pregnant women with common stenotic valvular lesions require maintenance of preload, afterload, and sinus rhythm while avoiding tachycardia and hypotension. According to the delivery of 12 parturients with moderate-severe aortic valve stenosis, 4 out of 12 patients with aortic valve area between 0.5 to 1 cm^2^and a peak gradient between 48 to 130 mmHg underwent elective cesarean delivery. Two of these cases had epidural anesthesia alone and 1 of them underwent general anesthesia plus intrathecal morphine using standard BP monitoring. One emergency cesarean delivery was performed under general anesthesia. It was concluded that carefully titrated regional analgesia was well tolerated in patients undergoing vaginal or cesarean delivery even in the presence of severe aortic stenosis without maternal or neonatal complications.^32^

While neuraxial anesthesia is used to be considered contraindicated in patients with *aortic valve stenosis*, recent literature states that neuraxial anesthesia may not be contraindicated in carefully selected patients, which supports using an epidural or CSE technique with careful slow titration to prevent rapid decrease in SVR under adequate monitoring to control hemodynamic status.^32^^,^^33^ Since the CSE technique combines the reliability of spinal anesthesia with the ability to titrate the anesthetic to the desired effect, the risk of maternal hypotension for cesarean delivery is decreased in patients with suspected significant cardiac disease. One successful CSE analgesia for labor followed by intrapartum cesarean delivery under general anesthesia for a patient with an aortic valve area of 0.7 cm^2^ and a maximum peak gradient of 169 mmHg was performed using intra-arterial BP monitoring and pulmonary artery catheter and followed postpartum for 48 hours in a high dependency unit.^34^ While standard monitoring is usually adequate for vaginal delivery, intra-arterial invasive BP monitoring and 24-48 hours postpartum close follow-up may facilitate peri- and postoperative management via a multidisciplinary approach. The American Heart Association stated that neuraxial block has typically been the preferred anesthesia technique for cesarean delivery even in patients with mWHO class III or IV lesions, though the choice of anesthetic technique should be individualized by the anesthesiologist.^1^

Pregnant women with PH are considered to have a very high maternal mortality rate varying between 30%-56%.^17^ A systematic review of parturients with PH (idiopathic, congenital heart disease associated, or other aetiology between 1997-2007 years) demonstrated that 78% of deaths occurred within the first month after delivery, and particularly primigravid parturients who received general anesthesia were at higher risk of death. Outcome predictors showed that patients receiving general anesthesia were 4 times more likely to die compared with patients receiving regional anesthesia (OR 4.37, 95% CI 1.28-16.5, *P* = .02).^35^ Since the maternal mortality rate in parturients with PH remains high, early advice on including contraception is required. If women with PH become pregnant, a multidisciplinary approach with consideration of advanced therapies is warranted. Improved outcomes in fully counseled women who chose to continue with pregnancy could be achieved with a tailored multiprofessional approach including early targeted therapy with early planned delivery and regional anesthetic techniques.^35^^-^^37^

According to a meta-analysis of pregnancy outcomes in women with significant valve disease, the mortality rate (3% vs. 2%), incidence of pulmonary edema (37% vs. 9%), arrhythmia (16% vs. 4%), stillbirth (4% vs. 2%), and preterm delivery (18% vs. 14%) in parturients with severe mitral stenosis was higher than that of severe aortic stenosis.^38^ In pregnant women with mitral valve disease, peripartum management should focus on maintaining stable hemodynamics while preventing factors that may precipitate pulmonary congestion or right ventricular overload. Antenatal therapy with diuretics and beta-blockers should be continued when clinically indicated to optimize volume status and control HR. A relatively slow HR should be maintained to prolong diastolic filling time and facilitate left ventricular preload. New-onset or pre-existing atrial fibrillation requires prompt management with appropriate rate or rhythm control, including electrical cardioversion when indicated, together with anticoagulation according to current recommendations. Aortocaval compression should be avoided by maintaining left uterine displacement throughout the peripartum period. The SVR should be preserved, and oxytocin should be administered cautiously, preferably as a slow infusion rather than a rapid bolus, because abrupt reductions in SVR accompanied by increases in CO may adversely affect maternal hemodynamics. Methylergometrine and 15-methyl prostaglandin F2α should be avoided because they may induce systemic hypertension, tachycardia, and increased pulmonary vascular resistance. Furthermore, adequate analgesia and meticulous avoidance of hypoxia, hypercarbia, and acidosis are essential to prevent increases in pulmonary vascular resistance and subsequent right heart strain. Anesthetic management for parturients with valvular heart disease for cesarean delivery revealed that regional anesthesia including CSE or slow titrating epidural was performed 62.1%.^39^^,^^40^ For CSE, intrathecal hyperbaric bupivacaine of 2.5-5 mg + 15-25 µg of fentanyl followed by epidural bolus of either 0.5% plain bupivacaine or 2% lidocaine was suggested.^41^

In contrast, chronic regurgitant lesions generally benefit from preservation of forward flow with avoidance of excessive increases in SVR. Women with pulmonary hypertension, severe ventricular dysfunction, Fontan circulation, or complex congenital heart disease remain at particularly high risk of peri-partum cardiovascular collapse and should be managed in tertiary referral centers by experienced multidisciplinary teams with immediate access to advanced hemodynamic support, including mechanical circulatory support when necessary.^36^

## Postpartum Management

Maternal cardiovascular risk does not end with delivery. The first 24-72 hours postpartum are characterized by substantial autotransfusion and redistribution of extracellular fluid, increasing the risk of heart failure, pulmonary edema, arrhythmias, and hypertensive complications. Consequently, women with moderate- or high-risk CVD should remain under close observation during the early postpartum period. Analgesia should be optimized to minimize sympathetic activation while facilitating early mobilization and respiratory function. Fluid administration should remain conservative, with careful assessment of urine output, oxygenation, and clinical signs of congestion. Ongoing multidisciplinary follow-up is essential to guide medication adjustment, thromboprophylaxis when indicated, breastfeeding counseling, and future pregnancy planning.^1^^,^^17^

### Postpartum Hemorrhage and Uterotonic Agents

In women with CVD who are at increased risk of postpartum hemorrhage (PPH), hemorrhagic shock may result in rapid hemodynamic deterioration due to the limited cardiovascular reserve. Active management of the third stage of labor, together with early prevention and prompt treatment of uterine atony, is of paramount importance. Potential cardiovascular adverse effects of first and second-line uterotonics must be carefully balanced against their benefits in controlling uterine atony and hemorrhage as presented in box 4.^42^^-^^44^

## Conclusions

For the anesthesiologists, successful peri-partum care depends on understanding lesion-specific cardiovascular physiology, anticipating hemodynamic changes associated with labor and delivery, and appropriate monitoring via implementing multidisciplinary management throughout pregnancy, delivery, and the postpartum period. Early risk stratification, collaborative multidisciplinary management for meticulous hemodynamic optimization, and individualized anesthetic planning are fundamental to improve maternal and neonatal outcomes.

## Figures and Tables

**Figure 1.**
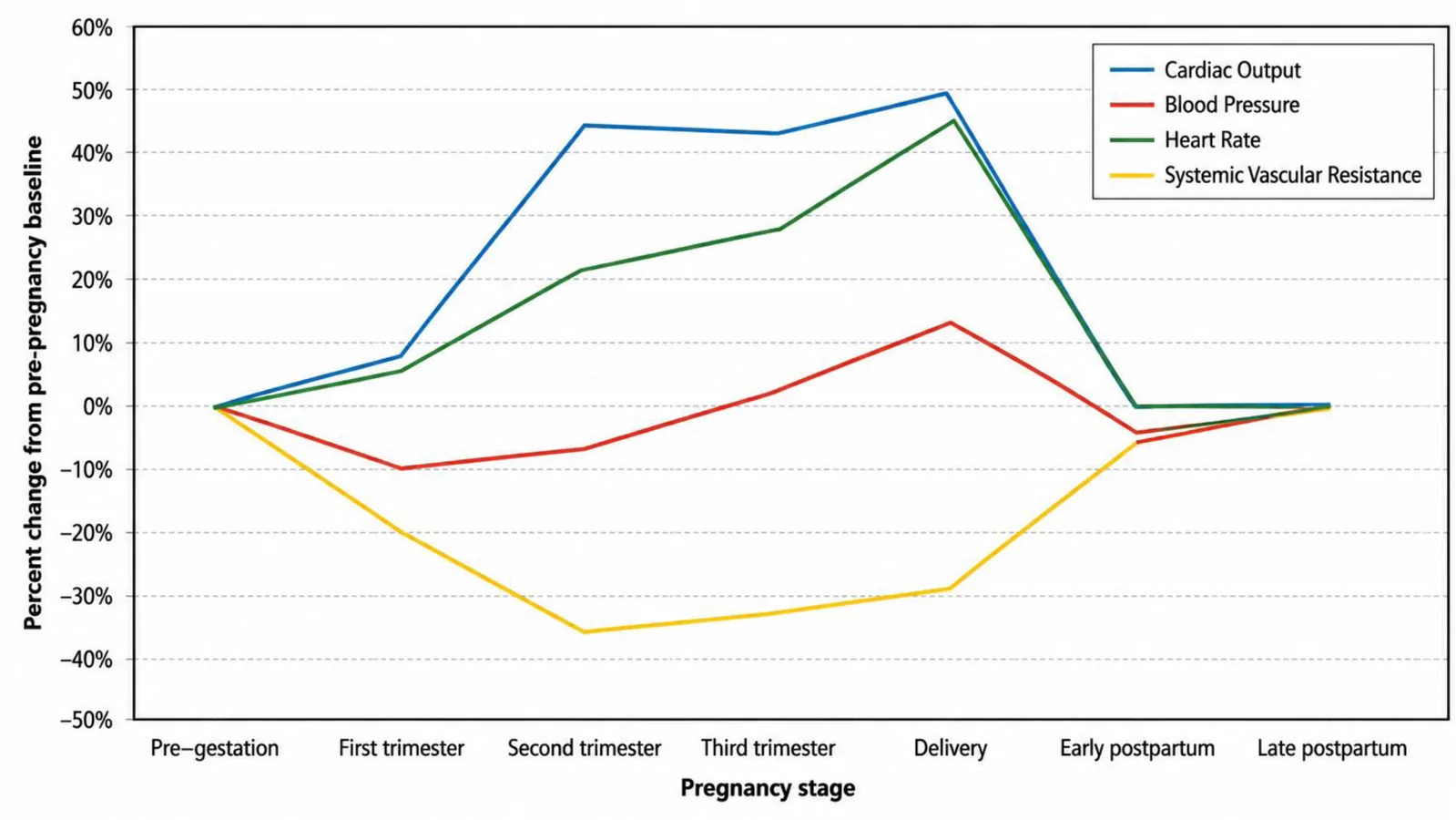
Cardiovascular physiological changes during pregnancy.

**Table 1.** Modified World Health Organization (mWHO) Classification of Maternal Cardiovascular Risk in Women with Pre-Existing Cardiovascular Disease (Adapted from the 2023 ESC Guidelines)

mWHO Risk	Representative Cardiac Lesions	Recommended Pregnancy Follow-up/Place of Delivery
Class I No detectable or only mildly increased maternal mortality or morbidity (2%-5% maternal cardiac event). Cardiology follow-up: once or twice during pregnancy.	• Uncomplicated mild pulmonary stenosis • Patent ductus arteriosus • Mitral valve prolapse • Successfully repaired simple lesions (atrial septal defect, ventricular septal defect, patent ductus arteriosus, anomalous pulmonary venous return) • Isolated atrial or ventricular ectopic beats	• Pre-pregnancy/pregnancy counselling • Follow-up at a local hospital • Delivery at a local hospital*
Class II Small increase in maternal mortality or moderate increase in morbidity (6%-10% maternal cardiac event). Cardiology follow-up: once each trimester.	• Unrepaired atrial or ventricular septal defect • Repaired tetralogy of Fallot or repaired coarctation of the aorta • Supraventricular arrhythmias • Turner syndrome without congenital heart disease	• Pre-pregnancy/pregnancy counselling • Pregnancy Heart Team consultation* • Follow-up at a local hospital • Delivery at a local hospital*
Class II-III Moderately increased maternal mortality or morbidity (11%-19% maternal cardiac event). Cardiology follow-up: once each trimester.	• Mild left ventricular dysfunction (LVEF > 45%) • Hypertrophic cardiomyopathy • Native or bioprosthetic valvular disease not included in mWHO Class I or IV (e.g., mild mitral stenosis, moderate aortic stenosis) • Marfan syndrome or another heritable thoracic aortic disease (HTAD) without aortic dilatation • Bicuspid aortic valve with aortic diameter < 45 mm • Repaired coarctation (non-Turner) • Atrioventricular septal defect	• Pre-pregnancy/pregnancy counselling • Pregnancy Heart Team consultation* • Follow-up at an appropriate-level hospital with availability of key Pregnancy Heart Team members according to the underlying cardiac disease • Delivery at an appropriate-level hospital*†
Class III Significantly increased maternal mortality or severe morbidity (20%-27% maternal cardiac event). Cardiology follow-up: every 1-2 months.	• Moderate left ventricular dysfunction (LVEF 30%-45%) • Previous peripartum cardiomyopathy without residual ventricular dysfunction • Mechanical prosthetic heart valve • Systemic right ventricle with preserved or mildly impaired ventricular function • Uncomplicated Fontan circulation • Unrepaired cyanotic congenital heart disease • Other complex congenital heart disease • Moderate mitral stenosis • Severe asymptomatic aortic stenosis • Moderate aortic dilatation (Marfan syndrome or other HTAD: 40-45 mm; bicuspid aortic valve: 45-50 mm; Turner syndrome: aortic size index 20-25 mm/m^2^; tetralogy of Fallot: <50 mm) • Ventricular tachycardia	• Pre-pregnancy/pregnancy counselling • Pregnancy Heart Team consultation* • Follow-up at an appropriate-level hospital • Delivery at an appropriate-level hospital*†
Class IV Pregnancy is contraindicated. Termination of pregnancy should be discussed. Extremely high maternal mortality or severe morbidity risk (>27% maternal cardiac event). Cardiology follow-up: at least monthly.	• Pulmonary arterial hypertension • Severe systemic ventricular dysfunction (EF < 30%, NYHA III-IV) • Previous peripartum cardiomyopathy with residual ventricular dysfunction • Severe mitral stenosis • Severe symptomatic aortic stenosis • Moderate-to-severe dysfunction of a systemic right ventricle • Severe aortic dilatation (Marfan syndrome or other HTAD > 45 mm; bicuspid aortic valve > 50 mm; Turner syndrome aortic size index > 25 mm/m^2^; tetralogy of Fallot > 50 mm) • Vascular Ehlers–Danlos syndrome • Severe recoarctation of the aorta • Complicated Fontan circulation	

ASI, aortic size index; EF, ejection fraction; HTAD, heritable thoracic aortic disease; LVEF, left ventricular dysfunction; NYHA, New York Heart Association.

*Pregnant women with a positive cardiac history, abnormal cardiovascular findings, or both should receive antenatal, intrapartum, and postpartum care in a hospital providing maternal care at Level II or higher, depending on the underlying cardiac lesion.

†**mWHO Risk Class III.** Pregnancy Heart Team should be available according to the underlying cardiac condition. E.g., a woman with a mechanical prosthetic heart valve should be managed in a centre with: (i) cardiology and maternal–fetal medicine services capable of weekly monitoring and adjustment of anticoagulation therapy; (ii) an obstetric unit with dedicated obstetric anesthesia services; and (iii) access to advanced cardiac care facilities and emergency cardiac surgery in the event of acute prosthetic valve thrombosis.^3^

‡**mWHO Risk Class IV.** Pregnancy generally is not advised because of the extremely high risk of maternal mortality and severe morbidity. If pregnancy occurs, in case of severe pulmonary hypertension should receive antenatal care and deliver in a tertiary referral centre providing maternal–fetal medicine expertise, dedicated obstetric and cardiac anesthesia services, specialist pulmonary hypertension care, and advanced heart failure therapies, including ventricular assist devices (VAD) and extracorporeal membrane oxygenation (ECMO).^3^

**Table 2.** Pregnancy Heart Team According to Modified WHO (mWHO) Maternal Cardiovascular Risk Classification Top of Form

Team Member	mWHO I	mWHO II	mWHO III-IV
Obstetrician	✓	✓	✓
Family physician/primary care physician	✓	✓	✓
Internist	✓	✓	✓
Cardiology consultation	As indicated	Recommended	Mandatory
Perinatologist (Maternal–Fetal medicine specialist)	—	✓	✓
Obstetric anaesthesiologist	—	Consider	✓
Cardiologist	—	Consider	✓
Congenital heart Disease specialist	—	As indicated	✓
Heart failure specialist	—	As indicated	✓
Cardiac electrophysiologist	—	As indicated	✓
Pulmonary hypertension specialist	—	As indicated	✓
Cardiac imaging specialist	—	As indicated	✓
Interventional cardiologist	—	As indicated	✓
Cardiac surgeon	—	As indicated	✓
Neonatologist	—	Consider	✓
Clinical Geneticist	—	As indicated	✓
Psychiatrist/psychologist	—	As indicated	✓
Clinical pharmacologist	—	As indicated	✓

WHO, World Health Organization.

## Data Availability

The data that support the findings of this study are available on request from the corresponding author.
